# Impaired CD8 T cell antiviral immunity following acute spinal cord injury

**DOI:** 10.1186/s12974-018-1191-8

**Published:** 2018-05-17

**Authors:** Diana M. Norden, John R. Bethea, Jiu Jiang

**Affiliations:** Department of Biology, Drexel University, 3245 Chestnut Street, Rm 415, Philadelphia, PA 19104 USA

**Keywords:** Spinal cord injury, Infection, Influenza virus, CD8 T cells

## Abstract

**Background:**

Spinal cord injury (SCI) disrupts essential neuroimmune communication, leading to severe immune depression. Previous studies confirmed immune dysfunction in mice with chronic SCI and following high thoracic level injury where sympathetic innervation of the spleen is disrupted. Here, we induced a mid-thoracic injury where integrity of the sympathetic response is maintained and investigated the antiviral T cell response to influenza virus after acute SCI.

**Methods:**

One week following a contusion SCI at thoracic level T9, mice were infected intranasally with influenza virus. Profiles of immune cell populations were analyzed before infection, and virus-specific CD8 T cell response was analyzed 7 days post-infection.

**Results:**

Following intranasal infection, injured mice had prolonged recovery and significant weight loss. Importantly, expansion and effector functions of virus-specific CD8 T cells were decreased in injured mice. The compromised CD8 T cell response was associated with inflammation and stress responses initiated after injury. Regulatory mechanisms, including increased regulatory T cells (Tregs) and upregulated PD-1/PD-L1, were induced following SCI. Furthermore, we show that increased corticosterone (CORT) levels can inhibit CD8 T cells and that blocking CORT in vivo following SCI enhances CD8 T cell antiviral responses.

**Conclusions:**

Our results show that mice with mid-thoracic SCI have impaired CD8 T cell function during the acute stage of injury, indicating that impaired antiviral responses occur rapidly following SCI and is not dependent on injury level.

## Background

Spinal cord injury (SCI) is a severe clinical condition affecting more than 1.3 million people in the USA [1]. In addition to impaired motor function, SCI leads to multiple organ dysfunction and complications and increased susceptibility to pathogen infection [2]. SCI and other disorders of the nervous system are known to induce severe immune depression resulting in increased risk of infections [1, 3–5]. Specifically, respiratory infections including Influenza A virus (IAV) and pneumonia occur frequently after SCI and are the leading cause of re-hospitalization and mortality in SCI patients [4–6]. Emergence of pandemic flu strains in the past decade have heightened the awareness that immune-compromised patients, such as those suffering from SCI, are more susceptible to a new strain of influenza virus [7]. Therefore, reducing complications from respiratory infections and understanding the mechanisms contributing to immune dysfunction are critical for improving the health and life span of SCI patients.

Intact neuroimmune communication is essential for mounting a proper immunological response to infections. Neural pathways that innervate peripheral organs regulate peripheral immunity through neural reflex circuits [8]. For example, immune cells express receptors for neurotransmitters including acetylcholine and norepinephrine (NE) which regulate immune activation [9]. Disrupted neuroimmune communication, such as that following SCI, results in a syndrome called CNS injury-induced immunodepression (CIDS), which is characterized by increased susceptibility to infections and worse neurological outcome [3]. Several groups, in addition to ours, have investigated the effects of SCI on immune dysfunction in models of acute and chronic SCI. Deficits in peripheral immunity with decreased antibody production by B cells [10, 11] and impaired cytokine expression by T cells [12, 13] have been reported in mouse models. Previously, these deficits have been attributed to sympathetic nervous system disruption. High thoracic (T3) injury disrupts essential sympathetic regulation of lymphoid organs and leads to impaired antibody synthesis and increased splenocyte apoptosis [10, 14]. These deficits may be related to elevated splenic norepinephrine (NE) levels since blocking NE signaling by β2 adrenergic receptor inhibition increased antibody production [10] and decreased splenic atrophy [14] after SCI.

In addition to altered sympathetic activity and elevated splenic NE, SCI also activates other stress responses. SCI can activate or dysregulate [15] the hypothalamic–pituitary–adrenal (HPA) axis leading to increased corticosterone (CORT) in the blood. Importantly, CORT levels were increased following both high- and mid-thoracic injures. For example, CORT levels increased rapidly after SCI [10, 16] and remained elevated 4 weeks after mid-thoracic SCI [14]. Because CORT has known anti-inflammatory effects [17], these findings suggest that CORT could interfere with immune function during acute and chronic SCI and that its effects are not limited to high thoracic injuries.

In our previous studies, chronic mid-thoracic (T9) contusion injury leads to impaired CD8 T cell function [12, 18]. We determined that chronically injured mice infected with H3N2 (X31) influenza virus were not able to mount an effective antiviral immune response. Following intranasal challenge with X31, SCI mice had significant mortality while non-injured mice exhibited no mortality [18]. Importantly, the increased mortality was associated with impaired generation and effector function of virus-specific CD8 T cells [18]. Interestingly, NE levels were elevated in the spleen of injured mice at this chronic timepoint [12]. However, mice with mid-thoracic injury also experience immune depression acutely after SCI [13] when NE levels are unchanged [10]. Therefore, we hypothesized that deficits in CD8 T cell function can occur independently of disrupted sympathetic regulation. To test this hypothesis, we investigated CD8 T cell antiviral responses to influenza virus after acute mid-thoracic injury. Our results show that mice with acute SCI have impaired CD8 T cell function, indicating that immune depression can occur independently of disrupted sympathetic splenic innervation and elevated NE.

## Methods

### Mice and SCI

Experiments were conducted in accordance with Drexel University Institutional Laboratory Animal Care and Use Committee. Adult (10 weeks old) female C57BL/6J wild-type (WT) mice and Clone-4 (HA_518–526_ TCR-Tg) mice were obtained from Jackson Laboratories. Contusion of the spinal cord was performed as previously described [18]. In brief, mice were anesthetized with a ketamine (100 mg/kg)-xylazine (10 mg/kg) cocktail. Using aseptic techniques, mice were subjected to laminectomy and subsequent contusion injury at thoracic level T9 using the Infinite Horizon Impactor at a predetermined force of 70 kDynes. Sham controls underwent anesthesia and a laminectomy at T9 without contusion injury. Mice were sutured and injected subcutaneously with 2-ml lactated Ringer’s solution to prevent dehydration and gentamicin (5 mg/kg) to prevent urinary tract infection. During recovery, mice received antibiotics and saline for 5 days and bladders were manually expressed twice per day [19].

### Influenza virus infection

To mimic clinically relevant respiratory infections in SCI patients, nasal application of influenza virus was used. Influenza virus subtype A/HKx31 (X31, H3N2) (Charles River) and A-Puerto Rico/8/34 (PR8, H1N1) were used for these studies. These two viruses were chosen because their infectivity efficiency in C57BL/6J mice. X31 is a reasserted virus which expresses the H3N2 surface protein of A/Hong Kong/1/1968 whereas PR8 expresses the surface protein hemagglutinin (HA) and neuraminidase (NA) of H1N1 subtype [20]. Both viruses contain six internal proteins common to the PR8 virus. X31 is intermediate in virulence compared to PR8; therefore, X31 was used for intranasal infection.

Seven days after injury, mice were anesthetized as described above and inoculated intranasally (i.n.) with 1 hemagglutination unit (HAU) X31 diluted in 20 μl sterile saline. This infection dose was chosen to limit mortality and allow for cellular studies based on pilot serial dose studies in uninjured mice. In a separate study, naïve mice were infected to generate effector cells for ex vivo analysis. Naïve mice were infected intravenously (i.v.) with 300 HAU PR8 diluted in 200 μl sterile saline [21]. This approach was chosen because PR8 is a more virulent compared to X31 and i.v. infection generates more effector cells in the spleen compared to i.n. infection [20, 21].

Following i.n. infection, mice were monitored daily up to 12 days for weight loss and euthanized if their body weight (BW) loss exceeded 25% of the preoperative weight (5 SCI mice, 0 uninjured mice). There was no difference between naïve mice and sham control in BW loss or CD8 T cell response (described below); therefore, naïve and sham were combined and represented as “Uninjured.”

### Drug administration

The glucocorticoid receptor antagonist mifepristone (Sigma-Aldrich) was dissolved in ethanol and diluted with sesame oil 1:10 prior to injection. Mice were injected i.p. with 30 mg/kg body weight daily starting 1 day after SCI [15].

### Cell preparation

Mice were sacrificed by CO_2_ asphyxiation followed by cervical dislocation, and the spleen and lungs were aseptically removed. The spleens were homogenized using the plunger of a 3-mL syringe (Becton Dickinson) through a 100-μm strainer and washed with RPMI-1640 medium (GenDEPOT). The lungs were minced using sterile scissors and digested in 5 mL RPMI containing 3 mg/mL Collagenase A (Sigma) and 0.15 mg/mL DNase I (Sigma) at 37 °C for 1 h. Digested lungs were homogenized using the plunger of a 3-mL syringe (Becton Dickinson) and filtered through a 40-μm strainer (EZFlow Cell Strainer, Foxx Life Sciences). Red blood cells were lysed using 0.83% ammonium chloride. Cells were washed and resuspended with complete RPMI (RPMI 1640, 5% FBS, 100 U/mL penicillin, 100 μg/mL streptomycin). The total number of live cells isolated was determined by trypan blue exclusion staining. The absolute number of cell populations were calculated based on the numbers of splenocytes/leukocytes from the lungs and the percentages of populations as determined by FACS.

### Ex vivo treatments and peptide stimulation

Following virus infection, splenocytes and leukocytes from the lungs were isolated and counted as above. Cells (10^6^ cells) were cultured with influenza viral peptide D^b^-PA_224–233_ and/or D^b^-NP_366–374_ (0.001 mg/mL) (AnaSpec) in the presence of IL-2 (50 U/mL) and GolgiStop for 4 h. Cells were then stained for surface markers and intracellular cytokines.

For ex vivo analysis of the effect of corticosterone (CORT) on CD8 effector T cells, naïve mice were infected intravenously (i.v.) with 300 HAU PR8. Splenocytes were isolated 7 days later and cultured with vehicle or 1 μm CORT (Sigma) for 1 h prior to addition of NP and polymerase acidic protein (PA) peptides. After 4 h of peptide treatment, cells were stained for surface markers and intracellular interferon gamma (IFNγ) production.

To determine the effect of CORT on naïve CD8 T cell proliferation capacity, splenocytes from Clone-4 mice were used because all CD8 T cells in this mouse are specific for viral HA peptide [22]. Using this approach, addition of HA peptide to splenocytes ex vivo leads to CD8 T cell activation and proliferation. Splenocytes were isolated and labeled with carboxyfluorescein succinimidyl ester (CFSE) [23]. Cells were incubated with 1 μM CORT overnight followed by HA peptide for 2 days. Cells were then stained for surface markers and profiled for proliferation based on CFSE labeling as described previously [23]. In brief, cells were gated for CFSE based on the untreated condition. Decreased CFSE (CFSE^low^) following stimulation indicates that cells have undergone proliferation.

### Flow cytometry

Cellular responses and the magnitude of the virus-specific CD8 T cell responses were analyzed by flow cytometry. Cell suspension aliquots were washed with staining buffer (1× PBS, 1% BSA) and immune cells were stained for using the following surface markers as described previously [12, 18]: CD8 T cells-CD8^+^, CD4 T cells-CD4^+^, B cells-B220^+^, Natural Killer cells-NK1.1^+^, Macrophages-CD11b^+^, LygC^high^, Neutrophils-CD11b^+^, Ly6G^+^, Dendritic cells-CD11c^+^, and MHCII^+^. Macrophage activation was determined by MHCII^+^ expression. PD1 and PDL1 surface expression was determined on CD8 T cells and macrophages, respectively. CD8^+^ effector cells were determined by gating on CD8 T cells followed by gating for CD44^+^CD62L^low^ expression. Antibodies were obtained from BD Pharmingen or eBioscience. Virus-specific CD8 T cells were quantified using tetramers (gated on CD8^+^ cells first, then on tetramer and CD44 to examine the activated virus-specific CD8 T cells). H-2D^b^-PA_224–233_ and H-2D^b^-NP_366–374_ tetramers were obtained from NIAID MHC Tetramer Core Facility (Atlanta, GA). For surface antibodies and tetramer labeling, cells were incubated for 30 min at 4 °C and then washed and fixed using 1% paraformaldehyde. Intracellular staining for IFNγ and granzyme B was performed using the Cytofix/Cytoperm kit (BD Pharmingen). Foxp3 staining was performed using the mouse Foxp3 buffer set (BD Pharmingen) according to the manufacturer’s protocol. Cells were analyzed using a FACS CanTo (Becton Dickinson), and data was analyzed using FlowJo software (Tree Star, Inc.).

### Statistics

All data was analyzed using one-way ANOVA with Tukey’s post hoc testing or Student’s unpaired *t* test when appropriate (IBM SPSS). Means and standard error of the mean (SEM) are reported throughout. Significance is set at *p* < 0.05.

## Results

### Prolonged recovery and decreased virus-specific CD8 T cell response after influenza virus infection in SCI mice

**Fig. 1:**
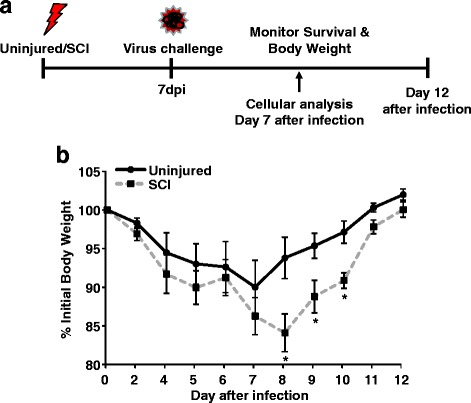
Increased body weight loss and prolonged recovery after intranasal X31 infection in SCI mice. Naïve/sham (uninjured) or SCI mice were infected i.n. with X31 7 days after injury. Body weight was monitored for 12 days, and percent decrease was determined. *p < 0.05 from uninjured by Student’s *t* test. Data represent six mice per group

Previous studies have confirmed immune depression in mouse models of chronic SCI [18]. Here, studies were performed to determine if the CD8 T cell response to influenza infection is impaired at an acute stage following SCI. Mice were challenged i.n. with influenza virus X31 7 days after T9 SCI and monitored for body weight loss through 12 days (Fig. 1a). One third of the SCI mice were removed from the study because of severe body weight loss (> 25% initial BW) after the infection, and all of the uninjured mice survived the challenge. It is important to note that SCI mice did have reduced weight compared to the uninjured mice 7 days after injury (day of infection). Therefore, the day of infection was used as the baseline for determining weight loss. As shown in Fig. 1b, the peak of weight loss occurred at 7 days post-infection in uninjured mice, while SCI mice continued to lose weight reaching the peak on day 8. Even with the reduced initial weight, the SCI mice still had significant weight loss on days 8, 9, and 10 following infection compared to uninjured mice (*p* < 0.05 for each time point). These data show that SCI mice have higher mortality and drastic weight loss following infection.

**Fig. 2:**
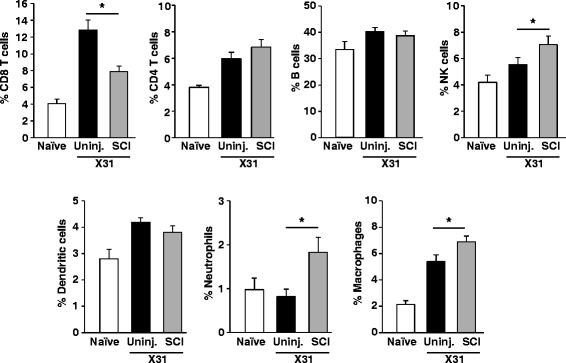
Impaired CD8 T cell accumulation and increased innate immune cells in the lung of SCI mice after virus infection. Naïve/sham (uninjured) or SCI mice were infected i.n. with X31 7 days after injury. Lungs were collected 7 days after infection, and the percentage of CD8 T cells, CD4 T cells, B cells, NK cells, dendritic cells, neutrophils, and monocytes was determined. *p < 0.05 from uninjured-X31 by one-way ANOVA. Data represent two independent experiments with at least four mice per group per experiment

The cellular response was analyzed at the peak of infection on day 7 to assess impaired functions that contribute to the prolonged recovery. First, we determined infiltration of immune cells into the lungs which is the target organ of viral replication following intranasal challenge. In uninjured mice, there was robust CD8 T cell recruitment to the lungs and this was significantly impaired after SCI (*p* < 0.001) (Fig. 2). In contrast to CD8 T cell recruitment, injured mice had enhanced recruitment of innate immune cells, including neutrophils, macrophages, and NK cells (*p* < 0.05 for all) (Fig. 2).

**Fig. 3:**
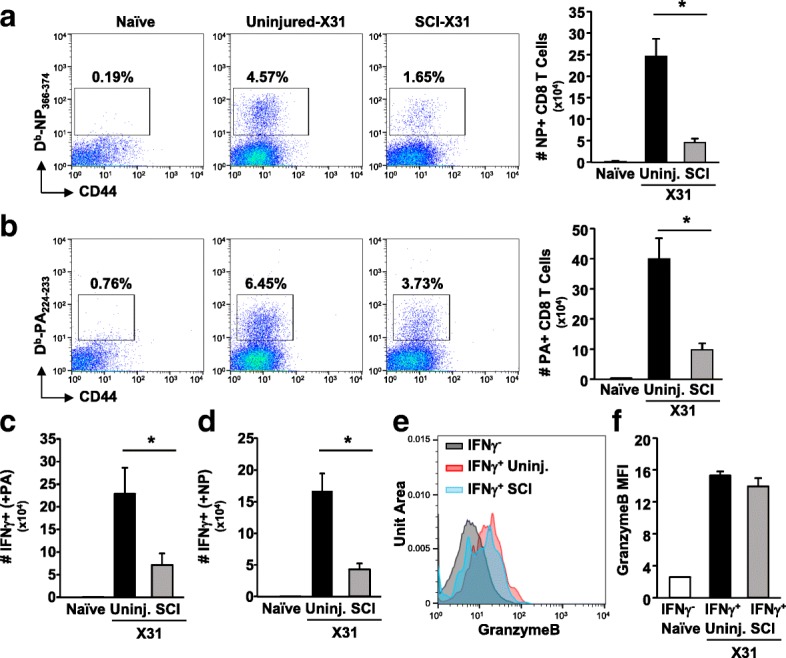
Attenuated virus-specific CD8 T cell expansion and function in SCI mice after virus infection. Mice received SCI and 7 days later were infected with X31 i.n. After 7 days, lungs were collected and CD8 T cells were analyzed. Representative dot plots of **a** NP and **b** PA tetramer staining. Cells were gated for CD8 T cells. The absolute number of **a** NP and **b** PA tetramer-positive CD8 T cells in the lung were determined. Cells were cultured ex vivo with NP or PA peptides. After 4 h, CD8 T cells were stained for intracellular IFNγ and granzyme B production. The absolute number of IFNγ-positive cells was determined after **c** PA and **d** NP stimulation. **e**, **f** MFI of granzyme B expression in IFNγ^−^ and IFNγ^+^ CD8 T cells. *p < 0.05 by one-way ANOVA. Data represent two independent experiments with at least four mice per group per experiment

In addition to decreased overall CD8 T cell infiltration into the lungs, SCI mice also had attenuated generation of virus-specific NP^+^ and PA^+^ CD8 T cells in the lungs. Figure 3a, b shows that both the percentage and absolute number of NP^+^ and PA^+^ CD8 T cells increased dramatically in uninjured mice. Although there were increased virus-specific CD8 T cells in the lung of SCI mice after infection, this was largely attenuated compared to uninjured mice (%NP: SCI vs uninjured, 3.87 ± 0.38 vs 1.88 ± 0.40, *p* < 0.003; %PA: SCI vs uninjured, 6.03 ± 0.63 vs 3.72 ± 0.55, *p* < 0.04; Fig. 3a, b). Following ex vivo stimulation with virus-specific peptide, CD8 T cells from infected uninjured mice had substantial INFγ production and this was significantly attenuated in SCI mice (NP *p* < 0.02, PA *p* < 0.01; Fig. 3c, d). Granzyme B expression was increased in IFNγ-producing CD8 T cells in both uninjured and SCI-infected mice, as measured by mean fluorescence intensity (MFI) (Fig. 3e). Interestingly, there was no difference in granzyme B production (MFI) on a per cell basis in specific CD8 T cells from uninjured and SCI mice (Fig. 3f). These data suggest that CD8 T cells in SCI mice are capable of cytotoxic functions and that the main impairment is due to decreased proliferation of specific CD8 T cells. Overall, these data show an impaired CD8 T cell response with diminished accumulation of specific CD8 T cells in the lung of SCI mice following viral infection.

**Fig. 4:**
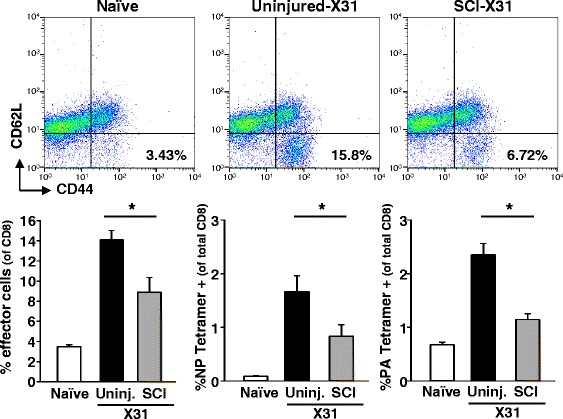
Attenuated CD8 T cell expansion and function in the spleen of SCI mice after virus infection. Mice received SCI and 7 days later were infected with X31 i.n. After 7 days, spleens were collected and splenocytes were isolated. Effector CD8 T cell expansion was determined by CD62L^low^/CD44^high^ expression. The percentage of tetramer-positive CD8 T cells was determined. *p < 0.05 by one-way ANOVA. Data represent two independent experiments with at least four mice per group per experiment

In addition to resident CD8 T cell responses to intranasal influenza infection, CD8 T cells can also be primed in lymphoid tissues including the spleen [24] before potentially infiltrating the lung. Therefore, we also determined the CD8 T cell response in the spleen of uninjured and SCI mice. After infection, uninjured mice had a large expansion of effector CD8 T cells (CD62^low^/CD44^high^) and this expansion of effector cells was significantly decreased in the spleen of injured mice (*p* < 0.003) (Fig. 4). Similar to the lung, the generation of virus-specific CD8 T cells was attenuated in the spleen of injured mice. The percentages of NP- and PA-specific CD8 T cells were lower in the spleen compared to the lung in both uninjured and SCI mice. However, specific CD8 T cells in the spleen were decreased in SCI mice compared to those in uninjured mice (*p* < 0.02) (Fig. 4). Overall, these data show that generation and effector function of CD8 T cells are impaired in the spleen of injured mice.

### SCI-induced inflammation increases regulatory pathways

**Fig. 5:**
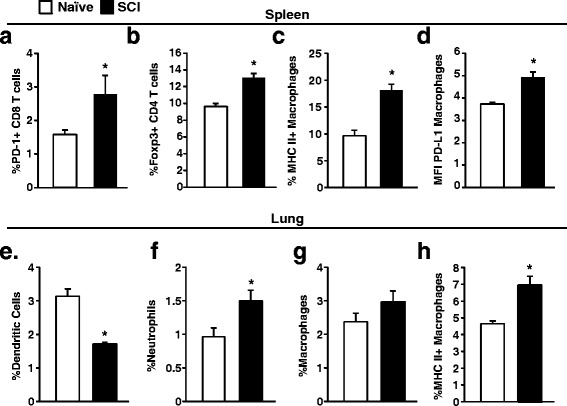
Increased expression of inhibitory molecules 7 days after SCI. Splenocytes were isolated 7 days after SCI and analyzed by flow cytometry for expression of regulatory pathways. **a** PD-1 expression was determined on CD8 T cells. **b** Foxp3 expression was determined in CD4 T cells. **c** MHC II and **d** PD-L1 was determined on macrophages. In the lung, the percentage of **e** dendritic cells, **f** neutrophils, **g** macrophages, and **h** MHC II^+^ macrophages was determined. *p < 0.05 by Student’s *t* test. Data represent six mice per group. *p < 0.05 by Student’s *t* test

SCI induces massive inflammation locally and systemically [2]. To evaluate possible SCI-induced changes in the immune system that could impair the response to virus, splenocytes were profiled 7 days after injury (before influenza challenge). In our previous studies [12], we showed that immune deficits after chronic SCI were dependent upon upregulation of the PD-1/PD-L1 pathway on immune cells. Here, we determined PD-1 expression on CD8 T cells and PD-L1 expression on macrophages 7 days after SCI. Similar to our previous studies, there was an increased percentage of CD8 T cells positive for PD-1 in injured mice (*p* < 0.05) (Fig. 5a). In addition, the MFI of PD1 on CD8 T cells was enhanced (PD1MFI: SCI vs uninjured, 1.58 ± 0.02 vs 1.88 ± 0.12, *p* < 0.04), indicating higher PD1 expression after injury. Macrophages in the spleen also had increased PD-L1 expression as measured by MFI (*p* < 0.001) (Fig. 5d). After SCI, macrophages were more activated with higher major histocompatibility complex class II (MHC II) expression (*p* < 0.001) (Fig. 5c). Not surprisingly, these data indicate that immune cells in the spleen have become activated from the injury. In addition, SCI mice had increased percentage of CD4 T cells that were positive for intracellular Foxp3, indicating more Tregs in the spleen after SCI (*p* < 0.001) (Fig. 5b).

We also investigated changes in immune cells in the lung 7 days after injury. There was no change in CD8 T cells, CD4 T cells, B cells, or NK cells in the lung following SCI. Interestingly, there was decreased dendritic cells in the lung after SCI which could have implications for decreased antigen presentation and decreased generation of specific CD8 T cells (*p* < 0.001) (Fig. 5e). There was a tendency (*p* = 0.08) for increased macrophages in the lung, and there was increased macrophage activation with higher MHCII expression in the lung of SCI mice (*p* < 0.002) (Fig. 5g, h). In addition, similar to that after infection, there were significantly increased neutrophils present in the lung of SCI mice (*p* < 0.02) (Fig. 5f). These results demonstrate that SCI leads to increased accumulation of neutrophils and activated macrophages in the lungs, and decreased dendritic cells, which could have implications for proper immune balance.

### CORT inhibits the function of flu-specific CD8 T cells

**Fig. 6:**
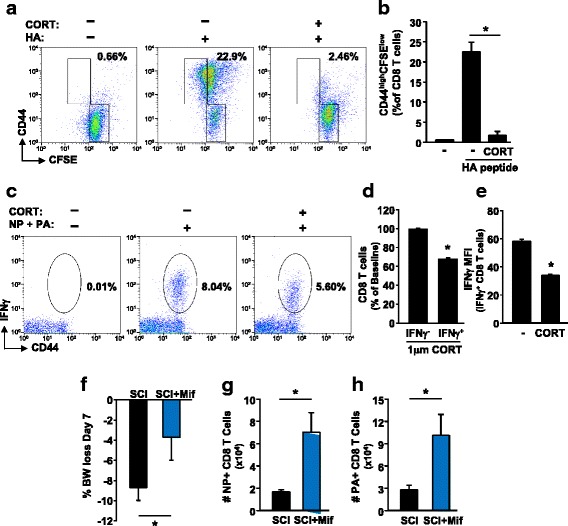
CORT inhibits the function of antigen-specific CD8 T cells. Splenocytes from Clone-4 mice were isolated, labeled with CFSE, and cultured ex vivo with vehicle or 1 μM CORT overnight. Viral peptide HA was added for 2 days and CD8 T cells were profiled based on CFSE/CD44 expression. **a** Representative dot plots of CFSE/CD44 expression. Cells were gated on CD8 T cells. **b** The percentage of CFSE^low^/CD44^high^ CD8 T cells was determined. Data represents duplicate treatments from three independent experiments. In a separate experiment, splenocytes were isolated 7 days after i.v. PR8 infection and cultured ex vivo with vehicle or 1 μM CORT for 1 h prior to addition of virus peptides NP and PA. After 4 h, CD8 T cells were stained for intracellular IFNγ production. **c** Representative dot plots of IFNγ production. Cells were gated for CD8 T cells. **d** The percentage decrease in IFNγ^−^ and IFNγ^+^ CD8 T cells was determined based on viable cell gate. **e** MFI for IFNγ in IFNγ^+^-specific CD8 T cells treated with vehicle or CORT treatment. Data represent splenocytes from six mice treated with each condition. Mice were treated with mifepristone (Mif) in vivo following SCI and through the infection. **f** Body weight loss and number of **g** NP and **h** PA-specific CD8 T cells was determined 7 days after infection. Data represent six mice per group. *p < 0.05 by Student’s *t* test

Next, we evaluated if increased CORT after SCI is a potential mechanism of impaired CD8 T cell responses by investigating if CORT attenuates effector CD8 T cell generation and activation. To investigate proliferation capacity of naïve-specific CD8 T cells, splenocytes from Clone-4 mice were used because all CD8 T cells in this mouse are specific for the viral HA_518–526_ peptide. HA stimulation induced the generation of effector cells (CD44^high^ and CFSE^low^ expression) (Fig. 6a), and this was significantly attenuated by CORT (Fig. 6a, b), indicating that CORT can inhibit the generation of activated effector CD8 T cells.

We also investigated the effect of CORT on effector CD8 T cell activation. Splenocytes were isolated 7 days after i.v. infection and cultured ex vivo with NP and PA peptides as well as vehicle or 1 μM CORT. CD8 T cell function/activation was measured using IFNγ production. IFNγ-producing CD8 T cells were observed upon peptide stimulation (Fig. 6c), while pretreatment with CORT significantly decreased the number of CD8 T cells producing IFNγ with about 30% (%IFNγ: vehicle vs CORT, 7.10 ± 0.79 vs 4.98 ± 0.58, *p* < 0.03, Fig. 6d). Importantly, there was only a decrease in the specific IFNγ-positive CD8 T cells and there was no decrease in non-specific IFNγ-negative CD8 T cells (Fig. 6d). This shows that CORT was having a direct effect on IFNγ production in virus-specific CD8 T cells. In addition, IFNγ production per cell was attenuated in the IFNγ-positive CD8 T cells as measured by MFI (*p* < 0.001) (Fig. 6e). These data indicate that CORT decreased both the number of IFNγ-positive cells and the level of IFNγ production per cell.

Last, we investigated whether increased CORT following SCI could interfere with antiviral immunity. Mice were treated in vivo with Mifepristone (Mif) to inhibit CORT signaling following injury and through the virus challenge. Following the virus challenge, mice treated with Mif lost significantly less weight compared to vehicle-treated mice (*p* < 0.05) (Fig. 6f). In addition, mice treated with Mif had increased number of flu-specific NP- (*p* < 0.05) and PA (*p* < 0.05)-positive CD8 T cells in the lungs compared to vehicle-treated mice (Fig. 6g, h). These data show that in vivo inhibition of CORT improved specific CD8 T cell expansion and decreased weight loss following infection.

## Discussion

Proper communication between the nervous system and peripheral immune system is necessary to maintain immune homeostasis and mount an immunological response to infection [8]. Peripheral neurons respond to infections through cytokine receptors and pattern recognition receptors [25]. In return, the nervous system itself can also activate immune cells. Neurogenic inflammation arises following release of inflammatory mediators from peripheral nerve terminals and has a direct effect on peripheral immune cells. These mediators include neuropeptides, neurotransmitters, and chemokines which can activate immune cells and facilitate immune cell recruitment, providing a positive feedback loop [9, 26]. Given the high innervation of the neuronal network, and the speed of neuronal transduction, neuroimmune communication can allow for rapid immune cell activation and mobilization [26]. Recent studies have investigated the significance of neuronal regulation of CD8 T cell function [18] and have identified immune dysfunction after neuronal damage, such as SCI. Following viral infection, expansion of virus-specific CD8 T cells is necessary to eliminate the infection. Therefore, restoring CD8 T cell function provides a therapeutic strategy for improving resistance to influenza virus after SCI. Understanding the mechanisms of immunological failure to respond to influenza and other infections in injured mice is highly significant and clinically relevant for injured patients who may suffer from life-threatening clinical complications from not only influenza infection but also other microbial infections.

In this study, we show that mice with acute SCI had exaggerated and prolonged weight loss following influenza virus infection. The peak of weight loss occurred at 7 days post-infection in uninjured mice, while SCI mice continued to lose weight though 8 days (Fig. 1b). In addition, several SCI mice had to be removed from the study because of severe weight loss when weight loss resulting from the injury was taken into consideration. We have previously shown that mice with chronic SCI had increased mortality following virus infection [18]. We surmise that a higher dose of virus would be completely lethal in injured mice; however, in this study a lower dose of virus was used to limit lethality and allow for further cellular studies. Seven days after infection, SCI mice had attenuated virus-specific CD8 T cells in both the lung and spleen (Figs. 3 and 4). There were both decreased total CD8 T cells and virus-specific CD8 T cells in the lung of SCI mice (Figs. 2 and 3), suggesting decreased proliferation of resident and specific CD8 T cells. Furthermore, we hypothesize that the attenuated response in the spleen contributes to decreased infiltration of specific CD8 T cells in the lung of SCI mice after infection, as other studies have suggested that splenic CD8 T cells can contribute to viral clearance in the lung [24]. Because neuronal signaling can be involved in immune cell recruitment [26], this lack of infiltration can be directly due to impaired neuroimmune communication following injury. Importantly, the impaired CD8 T cell response in lymphoid tissues can have implications for infections in various organs [13] in addition to systemic infections. These data indicate impaired CD8 T cell antiviral function acutely after SCI which leads to severe weight loss and prolonged recovery from infection.

Recent studies have begun to elucidate mechanisms of immunosuppression after SCI. For example, a recent study has implicated impaired regulation of the sympathetic-neuroendocrine adrenal reflex [15]. In addition, SCI-induced immune dysfunction has been shown to be due, in large part, to massive reorganization of the spinal sympathetic reflex circuit, e.g., the recruitment of glutamatergic interneurons, that results in increased sensitivity of this circuit [27]. These studies, however, only show immunosuppression following high thoracic (T3) injury. Importantly, our studies show impaired antiviral immune response following mid-thoracic (T9) injury. The mechanisms of immunosuppression following T9 injury are less understood. Here, we propose that the regulatory mechanism induced by SCI, such as increased Tregs, PD1, and CORT, in addition to an unbalanced immune response can interfere with antiviral immunity. However, more detailed mechanistic studies are required to fully understand how SCI leads to impaired antiviral immunity following T9 injury.

SCI causes massive inflammation locally and also systemically [2, 28]. Inflammatory cells are released into the blood stream and can then infiltrate both the injured spinal cord and also secondary organs, including the lung. Here, we show infiltration of neutrophils and macrophages into the lung following SCI (Fig. 5). This is important because exaggerated neutrophil accumulation can impair CD8 T cell proliferation and activation [29, 30] and thus potentially impair viral clearance. Furthermore, there was an unbalanced cellular response to infection in the lung of SCI mice. SCI mice had decreased accumulation of CD8 T cells while innate immune cells, including macrophages and neutrophils, were exaggerated (Fig. 2). It is unknown if this unbalance in immune cells contributes to impaired CD8 T cell function. Innate immune cells like neutrophils, macrophages, and dendritic cells are important for activating and recruiting CD8 T cells during infections. However, altered activation state and function following SCI may interfere with their normal roles during infection. Further studies are needed to determine whether chemotactic signaling by innate immune cells is impaired following SCI. The reason why neutrophils accumulate in the lung after SCI is still unknown, but may be related to leaked gut microbiota that has translocated into lung tissue as recently reported [31]. Overall, our finding of increased neutrophils in the lung of SCI mice, both before and after infection, could have serious implications for pulmonary function [32] and antiviral responses.

Cellular profiling showed upregulation of several immunomodulatory pathways (PD-1/PD-L1, Tregs) in the spleen of injured mice (Fig. 5). Although the mechanism of how PD1 and Tregs are induced following SCI is still unknown, it is understood that these pathways are upregulated to counteract the systemic inflammatory response in order to limit further spread of inflammation at the injury site and also protect against autoimmunity [2]. For example, Tregs can promote tissue remodeling and depletion of Tregs during SCI worsened outcome [33]. The role of PD1 expression on T cells during recovery from SCI has not been studied in detail; however, PD1 expression on macrophages within the injured spinal cord was important for limiting inflammation and improving recovery [34]. Although PD1 and Tregs have roles in healing and recovery, they can also interfere with antiviral immune function during infection. We have previously shown that PD1 upregulation during chronic SCI decreases CD8 T cell activation [12]. In addition, Tregs suppress antiviral immunity which can enhance viral replication [35]. Overall, it is possible that the regulatory mechanisms set in motion following injury lead to attenuated responses to influenza challenge. Given the importance of the spleen as a lymphoid organ, the upregulation of these pathways could contribute to the impaired response to both systemic [13] and respiratory infections.

Deficits in peripheral immunity could also be attributed to sympathetic nervous system disruption and enhanced splenic NE signaling. Adrenergic nerve terminals in the spleen and liver regulate NE release [9]; however, this regulation becomes impaired following injury, leading to enhanced NE levels and decreased immune activation. Previous studies have shown that T3 transection which completely disrupts essential sympathetic regulation of lymphoid organs leads to increased splenocyte apoptosis and impaired immune function that was dependent on NE signaling [10, 14]. In addition, we have previously shown that NE increases PD1 expression and directly decreases activation of CD8 T cells [12]. However, NE levels are unchanged early after T9 injury [10, 14]. In addition to neurotransmitters like NE, altered neuroimmune communication and increased neuroinflammation can also increase levels of stress-related hormones, including CORT. SCI activates or dysregulates the HPA axis [15] leading to increased CORT in the blood. For example, CORT levels increased rapidly after SCI [10, 16] and remained elevated 4 weeks after SCI in both T3 and T9 injured mice [14]. Increased CORT is relevant for NE signaling because CORT can increase β2-adrenergic receptor expression and ligand affinity on immune cells [36, 37]. Therefore, it is possible that CORT could interfere with immune function after acute T9 contusion through enhanced NE sensitivity which decreases CD8 T cell activation.

In this study, we investigated the direct effect of CORT on CD8 T cell proliferation and activation. CORT significantly attenuated proliferation of specific CD8 T cells following activation ex vivo. Furthermore, CORT directly decreased CD8 T cell activation. Effector CD8 T cells treated with CORT had decreased IFNγ production following ex vivo stimulation. Therefore, we believe that systemic changes in immune function and increased stress responses have a negative effect on viral clearance. In addition to increasing NE sensitivity, there are two known mechanisms by which CORT can attenuate the CD8 T cell response. First, CORT has been shown to decrease the ability of antigen-presenting cells to induce proliferation and activation of CD8 T cells [38, 39]. In addition, CORT may decrease IFNγ production directly in the CD8 T cells as T cells also express glucocorticoid receptors [40]. Importantly, following in vivo inhibition of CORT, SCI mice had increased expansion of specific CD8 T cells and mice treated with mifepristone had decreased weight loss compared to vehicle-treated mice. Overall, our data indicate that CORT can have a direct effect on activation of specific CD8 T cells.

A recent study has identified adrenal reflex dysfunction as a contributor to immunesuppression following high thoracic injury [15]. Preventing a spike in CORT following injury while still maintaining normal levels of CORT improved immune cell function and prevented the onset of spontaneous pneumonia [15]. In this study, however, none of the mice with mid-thoracic injury developed pneumonia, indicating a level-dependent susceptibility to pneumonia infection. In our study, we show impaired CD8 T cell response to viral infection following mid-thoracic injury. Therefore, the innate immune response necessary to clear bacterial infection may be more level-dependent compared to CD8 T cell responses necessary for viral infections, and impaired CD8 T cell function could be a common mechanism of immune dysfunction following both high- and mid-thoracic injuries. It is also possible that mice with mid-thoracic injury would have decreased clearance of pneumonia if infected, as compared to generation of spontaneous infections. Further studies are necessary to identify common mechanisms of immune dysfunction following different levels of SCI and the role of CORT in regulating CD8 T cell function.

## Conclusions

In summary, our study shows impaired CD8 T cell antiviral responses to influenza virus following acute SCI that lead to prolonged recovery from infection. Here, we used a biorelative approach of intranasal inoculation to investigate respiratory infections, but the data presented suggest systemic deficits in CD8 T cell function. The regulatory mechanisms set in motion following injury may be one potential mechanism that leads to attenuated responses to influenza challenge. In addition, the systemic inflammatory cascade and stress responses initiated by SCI can cause severe immune depression and multiple organ dysfunction [2]. Given the severe condition of SCI patients, understanding the mechanisms of immune dysfunction after SCI is critical to avoid infections and other serious complications. Our results indicate that immune depression can occur independently of disrupted sympathetic splenic innervation and elevated NE and may be more dependent on increased CORT levels. This finding will open up new avenues for investigating immune cell function following SCI.

## Abbreviations

BW: Body weight
CFSE: Carboxyfluorescein succinimidyl ester
CIDS: CNS injury-induced immune depression
CNS: Central nervous system
CORT: Corticosterone
HA: Hemagglutinin
HAU: Hemagglutination unit
HPA: Hypothalamic–pituitary–adrenal
i.n.: Intranasal
i.v.: Intravenous
IAV: Influenza A virus
IFNγ: Interferon gamma
MFI: Mean fluorescent intensity
MHC II: Major histocompatibility complex class II
NA: Neuraminidase
NE: Norepinephrine
NK: Natural killer
PA: Polymerase acidic protein
SCI: Spinal cord injury
SEM: Standard error of the mean
Treg: Regulatory T cell
WT: Wild-type
